# Supplementary material for: Impact of Fermentable Fibres on the Colonic Microbiota Metabolism of Dietary Polyphenols Rutin and Quercetin
Source: Int J Environ Res Public Health. 2019 Jan 21;16(2):292. doi: 10.3390/ijerph16020292 (PMC6352267; doi:10.3390/ijerph16020292)
Supplement: Supplementary file 1 [file ijerph-16-00292-s001.pdf]

Table S1. PA production during rutin incubation in in vitro fecal batch cultures for each participant.

	V-3	V-4	V-5	V-6	V-7	V-8	V-9	V-10	V-11	V-12
**4-OHBA (μg/mL)**										
**R-0 h**		0.00	0.00							
**R-2 h**		0.06	0.00							
**R-6 h**		0.06	0.00							
**R-24 h**		0.05	0.02							
**PAA (μg/mL)**										
**R-0 h**	0.57	0.15	0.35	0.15	0.40	0.79	0.29	0.23	0.42	0.00
**R-2 h**	0.67	0.73	0.87	0.58	0.38	0.57	0.43	1.22	1.90	2.63
**R-6 h**	1.07	0.47	1.56	0.42	0.64	1.03	1.56	5.66	5.08	1.66
**R-24 h**	4.64	2.37	11.62	3.59	1.25	4.16	6.31	4.94	6.27	10.83
**3-OHPAA (μg/mL)**										
**R-0 h**	0.00	0.00	0.00	0.00	0.00	0.00	0.00	0.00	0.00	0.00
**R-2 h**	0.00	0.00	0.00	0.00	0.00	0.00	0.00	0.00	0.00	0.00
**R-6 h**	0.00	0.00	0.10	0.20	0.04	0.00	0.00	0.09	0.06	0.09
**R-24 h**	0.06	0.08	0.52	2.40	0.07	0.00	1.89	0.54	1.97	5.03
**4-OHPAA (μg/mL)**										
**R-0 h**	0.08	0.04	0.00	0.01	0.07	0.28	0.04	0.08	0.12	0.84
**R-2 h**	0.21	0.18	0.15	0.14	0.22	0.21	0.19	1.97	2.13	2.41
**R-6 h**	0.50	0.08	0.23	0.12	0.25	0.74	0.20	0.69	1.79	1.72
**R-24 h**	0.49	2.18	0.65	0.22	0.55	1.18	0.10	0.11	1.30	1.16
**3,4- diOHPAA (μg/mL)**										
**R-0 h**	0.00	0.00	0.00	0.00	0.00	0.00	0.00	0.00	0.00	0.00
**R-2 h**	0.44	0.14	0.19	0.24	0.36	0.32	1.69	1.15	1.13	1.37
**R-6 h**	1.68	0.01	1.53	3.15	0.82	4.15	4.75	2.51	2.11	8.90
**R-24 h**	5.27	0.46	7.31	0.32	5.24	12.36	0.02	0.08	0.02	0.03
**3-OHPPA (μg/mL)**										
**R-0 h**	0.10	0.02	0.00	0.31	0.16	0.38	0.30	0.06	0.27	0.59
**R-2 h**	0.18	0.22	0.26	0.70	0.32	0.27	0.42	0.20	0.52	0.37
**R-6 h**	0.23	0.03	0.23	0.28	0.21	0.50	0.67	1.08	1.54	0.72
**R-24 h**	0.23	0.05	0.77	0.40	0.56	0.79	0.37	0.10	0.27	0.78
**4-OHPPA (μg/mL)**										
**R-0 h**	0.00	0.00	0.00	0.06	0.00	0.15	0.00	0.00	0.00	0.00
**R-2 h**	0.08	0.00	0.18	0.30	0.18	0.18	0.20	0.24	0.80	0.76
**R-6 h**	0.00	0.00	0.43	0.07	0.09	0.27	1.01	0.24	1.88	0.27
**R-24 h**	0.00	0.33	0.09	0.13	0.58	2.27	0.06	0.02	0.04	0.37

Values are mean of duplicate measurements at 0, 2, 6 and 24 h for rutin only incubations. R: rutin, h: hour.
